# Characterization of Four Novel dsRNA Viruses Isolated from *Mucor* *hiemalis* Strains

**DOI:** 10.3390/v13112319

**Published:** 2021-11-21

**Authors:** Tünde Kartali, Ildikó Nyilasi, Sándor Kocsubé, Roland Patai, Tamás F. Polgár, Nóra Zsindely, Gábor Nagy, László Bodai, Zoltán Lipinszki, Csaba Vágvölgyi, Tamás Papp

**Affiliations:** 1 Department of Microbiology, Faculty of Science and Informatics, University of Szeged, 6726 Szeged, Hungary; nyilasiildi@gmail.com (I.N.); shigsanyi@gmail.com (S.K.); zsindizsn@yahoo.com (N.Z.); csaba@bio.u-szeged.hu (C.V.); 2 Neuronal Plasticity Research Group, Institute of Biophysics, Biological Research Centre, 6726 Szeged, Hungary; patai.roland@brc.hu (R.P.); polgar.tamas@brc.hu (T.F.P.); 3 Theoretical Medicine Doctoral School, University of Szeged, 6722 Szeged, Hungary; 4 Department of Biochemistry and Molecular Biology, Faculty of Science and Informatics, University of Szeged, 6726 Szeged, Hungary; gbrngy@gmail.com (G.N.); bodai@bio.u-szeged.hu (L.B.); 5 MTA SZBK Lendület Laboratory of Cell Cycle Regulation, Institute of Biochemistry, Biological Research Centre, Eötvös Loránd Research Network (ELKH), 6726 Szeged, Hungary; lipinszki.zoltan@brc.hu; 6 MTA-SZTE Fungal Pathogenicity Mechanisms Research Group, Hungarian Academy of Sciences and Department of Microbiology, University of Szeged, 6726 Szeged, Hungary

**Keywords:** mycovirus, dsRNA, virus particle, *Totivirus*, *Victorivirus*, Mucorales

## Abstract

We previously screened the total nucleic acid extracts of 123 *Mucor* strains for the presence of dsRNA molecules without further molecular analyses. Here, we characterized five novel dsRNA genomes isolated from four different *Mucor* *hiemalis* strains with next-generation sequencing (NGS), namely Mucor hiemalis virus 1a (MhV1a) from WRL CN(M) 122; Mucor hiemalis virus 1b (MhV1b) from NRRL 3624; Mucor hiemalis virus 2 (MhV2) from NRRL 3616; and Mucor hiemalis virus 3 (MhV3) and Mucor hiemalis virus (MhV4) from NRRL 3617 strains. Genomes contain two open reading frames (ORF), which encode the coat protein (CP) and the RNA dependent RNA polymerase (RdRp), respectively. In MhV1a and MhV1b, it is predicted to be translated as a fusion protein via -1 ribosomal frameshift, while in MhV4 via a rare +1 (or−2) ribosomal frameshift. In MhV2 and MhV3, the presence of specific UA**A****UG** pentanucleotide motif points to the fact for coupled translation termination and reinitialization. MhV1a, MhV2, and MhV3 are part of the clade representing the genus *Victorivirus*, while MhV4 is seated in *Totivirus* genus clade. The detected VLPs in *Mucor* strains were from 33 to 36 nm in diameter. Hybridization analysis revealed that the dsRNA molecules of MhV1a-MhV4 hybridized to the corresponding molecules.

## 1. Introduction

Despite the increasing number of the sequenced mycoviruses registered in the NCBI (National Center for Biotechnology Information) database, we still have sporadic information about fungal viruses. [1]. A vast majority of the described mycoviruses have linear double-stranded RNA (dsRNA) genomes [2,3], and the mycoviral infections are mostly cryptic, as their presence is usually symptomless in their hosts [2,4]. Viruses in the *Totiviridae* family generally contain a non-segmented and bicistronic genome between 4.6–7.0 kbp in length. The two frequently overlapping open reading frames (ORFs) encode the capsid/coat protein (CP) and the RNA-dependent RNA polymerase (RdRp) [2,5,6]. Currently, five genera are discriminated within the family, among which the genera *Totivirus* and *Victorivirus* contain mycoviruses [2]. It is worth mentioning that several mycoviruses in this family cannot be classified into any of the presently accepted genera and are considered as unclassified totiviruses [7]. Although mycoviruses have been identified in almost all major fungal phyla, their presence has been pointed at mostly in Ascomycota and Basidiomycota species [2,3]. This tendency seems to be changing lately since an increasing number of mycoviruses are identified from the so-called early-diverging fungal lineages, including Mucoromycota. In a recent study, Myers et al. (2020) found 27.1% of the 107 Mucoromycota fungi screened by cellulose chromatography or transcriptome-mining positive for mycoviruses [8]. Previously, virus-like particles (VLPs) were detected in five *Rhizopus* isolates [9], a geminivirus-like ssDNA virus was described in *Mucor racemosus* [10], and a (−) ssRNA virus was detected in *Mucor irregularis* [11]. Recently, four viruses with dsRNA genomes were detected and characterized in *Umbelopsis ramanniana* [7], and two (+) ssRNA narnaviruses were reported from *Rhizopus microsporus* [12].

Earlier, Vágvölgyi et al. (1998) screened the total nucleic acid extracts of 123 *Mucor* strains representing 18 species for the presence of dsRNA molecules [13]. DsRNA elements and isometric, non-enveloped VLPs were detected in five strains (i.e., *M. corticolus* NRRL 3616 and NRRL 3617, *M. hiemalis* NRRL 3624, *M. mucedo* WRL CN(M) 122, and *M. aligarensis* SZMC ) without further molecular studies. The aim of the present study was to re-examine these strains and provide a more detailed characterization of their dsRNA elements. *M. corticolus* and *M. hiemalis* are presently classified as *M. hiemalis* f. *corticola* and *Mucor hiemalis* f. *hiemalis*, respectively. The *M. aligarensis* strain was not available, and the strain WRL CN(M) 122 proved to be *Mucor hiemalis* f. *hiemalis* based on ITS sequencing. Thus, we used these four strains to sequence and characterize viruses in *Mucor hiemalis* strains.

## 2. Materials and Methods

### 2.1. Fungal Strains and Cultivation

Two *Mucor hiemalis* f. *hiemalis* (i.e., WRL CN(M) 122 and NRRL 3624) and two *M. hiemalis* f. *corticola* (i.e., NRRL 3616 and NRRL 3617) strains (Table 1) were tested for the presence of dsRNA molecules. Each strain showed the phenotype characteristic to the corresponding species and subtaxon without any apparent abnormities. The strains were maintained on malt extract agar slants (0.5% malt extract, 0.5% yeast extract, 1% glucose, 2% agar) at 4 °C. Mycelia for virus particle and dsRNA purification were grown in yeast extract−glucose broth (1% glucose, 0.5% yeast extract) at 25 °C for 4 days on an orbital shaker (150 rpm).

### 2.2. Isolation of dsRNA Molecules

To detect the dsRNA elements, a variation of the lithium chloride-based total nucleic acid extraction method of Leach et al. (1986) was used [14]. 

For next-generation sequencing (NGS) and “full-length amplification of cDNAs” (FLAC), CF-11 cellulose chromatography was used to purify dsRNA elements from total nucleic acid extracts purified with LETS buffer extraction. The purification was done according to the method of Morris and Dodds (1979) [15] with minor modifications [7]. 

All dsRNA samples were separated by electrophoresis on 0.8% agarose/TAE (40 mM Tris/acetic acid, 1 mM EDTA, pH 7.6) horizontal gels. Nucleic acids were visualized by UV fluorescence after ethidium bromide (0.5 μg/mL) staining. The relative sizes of the dsRNA molecules were estimated using GeneRuler 1 kb DNA ladder (Thermo Scientific,Vilnius, Lithuania) as size standards. The nature of the detected dsRNA elements was confirmed by their resistance to DNase I (Thermo Scientific, Vilnius, Lithuania) and S1 nuclease (Thermo Scientific, Vilnius, Lithuania) digestions, which were carried out according to the manufacturers’ recommendations.

### 2.3. Purification and Examination of the Virus Particles

Virus particles were purified from 30 g frozen mycelium according to the method of Lot et al. (1972) [16]. The pellet was resuspended in 150 μL borate buffer (5 mM boric acid, 1.475 mM sodium tetraborate, 0.5 mM EDTA) and then placed on a discontinuous sucrose density gradient (10% to 40% (*w*/*v*) sucrose in PBS) for ultracentrifugation at 70,000× *g* for 2 h at 4 °C in a swinging-bucket rotor (Beckman Coulter MLS-50). VLPs were collected from the ~35% sucrose layer, dialyzed against PBS (16 h at 4 °C), and pelleted by ultracentrifugation at 144,000× *g* for 2 h at 4 °C in a fixed-angle rotor (Beckman Coulter TLA-110). The VLP-containing pellet was resuspended in 30 μL PBS and analyzed by transmission electron microscopy. Purified virus particles were analyzed with a JEM-1400 Flash transmission electron microscope (JEOL) to identify the morphological characteristics of the particles. We used the method described in [7] to obtain the negatively stained samples, which were systematically screened at 30,000× magnification to localize the presence of the virus particles on the grid. Afterwards, the particles were recorded at 60,000× magnification with a 16 MP Matataki Flash scientific complementary metal–oxide–semiconductor (sCMOS) camera (JEOL).

### 2.4. Sequencing Library Generation, NGS Sequencing and Bioinformatic Analysis of Sequencing Data

RNA sequencing libraries were generated from 80 ng viral genomic RNA samples using NEBNext Ultra II Directional RNA Library Prep Kit for Illumina (New England Biolabs, San Diego, USA) with NEBNext Multiplex Oligos for Illumina (New England Biolabs, San Diego, USA) following the manufacturer’s protocol for use with purified mRNA or rRNA depleted RNA. This includes, in short, fragmentation of RNA followed by first- and second-strand cDNA synthesis, cDNA end repair, adaptor ligation, and enrichment of adaptor ligated library fragments by limited PCR. Sequencing libraries were validated and quantified with a 2100 Bioanalyzer (Agilent, Santa Clara, US) instrument using Agilent DNA 1000 Kit; then, after pooling and denaturing, they wer sequenced in an Illumina MiSeq instrument with MiSeq Reagent kit V3-150 generating 2 × 75 bp paired-end sequence reads.

FASTQ files were generated with GenerateFASTQ 1.1.0.64 application on Illumina BaseSpace. Sequence quality checks and adapter trimming was done using TrimGalore with parameters: —paired —length 36 —q 20. To filter out host sequences, we aligned sequence reads to reference genome assemblies of *Mucor* species (*M. mucedo*, *M. hiemalis,* or *M. corticolus*) available from the Joint Genome Institute (JGI) website (https://jgi.doe.gov/, accessed on 26 October 2021) using bowtie2 [17] (with parameters: —very—sensitive—local) and kept only those reads that did not align to the host genomes. As a secondary filter, we aligned the filtered reads to a subject sequence database generated from *Mucor*-specific sequences available at National Center for Biotechnology Information (NCBI) using BLASTn (https://blast.ncbi.nlm.nih.gov/Blast.cgi (accessed on 26 October 2021).) and used only in further analysis those sequence reads that did not align. After read filtering, we performed genome assembly using SPAdes with –careful parameter. The sequencing depth of the viral genome assemblies was 442, 634, 601, 355, and 297 for WRL CN(M) 122, NRRL 3624, NRRL 3617, NRRL 3616 scaffold 1, and NRRL 3616 scaffold 2, respectively. The identified sequences were deposited to European Nucleotide Archive (ENA; accession numbers: HG993402-HG993409, OU374593-OU374594) and to NCBI database (accession no.: OK157910).

### 2.5. cDNA Synthesis and Sequencing of the dsRNA Molecules 

To determine the 5′ and 3′ termini of the genomes by amplification of cDNAs from the dsRNA templates, the FLAC technique [18] was used. Ligation of the PC3-T7 loop primer (5′-pGGATCCCGGGAATTCGGTAATACGACTCACTATATTTTTATAGTGAGTCGTATTA-OH-3′; [19] to the purified dsRNA fragments, the denaturation of the primer-ligated dsRNAs, and cDNA synthesis reaction were performed as described previously [20]. Amplification of the cDNA was performed using 0.5 µM PC2 primer (5′- CCGAATTCCCGGGATCC-3′; [19] and 0.5 µM specific primer (Supplementary Table S2) and 1 unit of the Phusion High-Fidelity DNA Polymerase (Thermo Scientific, Vilnius, Lithuania). The PCR was incubated in a MJ Mini 48-Well Personal Thermal Cycler (Bio-Rad, Hercules, CA, USA) at 98 °C for 1 min, followed by 35 cycles of 98 °C for 10 s, 66 °C for 30 s, and 72 °C for 4 min and a final elongation at 72 C for 10 min. PCR products were purified from the agarose gel with the Zymoclean Large Fragment DNA Recovery Kit (Zymo Research, Irvin, CA, USA). Purified products were then cloned into the pJET1.2/Blunt vector (CloneJET PCR Cloning Kit, Thermo Scientific, Vilnius, Lithuania). Sequences of the inserts were determined by the Eurofins Genomics Germany GmbH.

### 2.6. Sequence and Phylogenetic Analysis

The sequences were subjected to BLAST searches (http://blast.ncbi.nlm.nih.gov/Blast.cgi (accessed on 26 October 2021).) in the NCBI nucleic acid and protein databases. Putative proteins were predicted and analyzed using the tools of the Expasy Bioinformatics Resource Portal (https://www.expasy.org/ (accessed on 26 October 2021).). The DotKnot program (https://dotknot.csse.uwa.edu.au/ (accessed on 26 October 2021).) [21] was used to predict possible RNA H-type pseudoknots. To visualize the possible pseudoknots, the PseudoViewer program (http://pseudoviewer.inha.ac.kr/ (accessed on 26 October 2021).) [22] was used. Molecular weights of the identified proteins were predicted with the Protein Molecular Weight program (https://www.bioinformatics.org/sms/prot_mw.html (accessed on 26 October 2021).). To determine the similarity of amino acid sequences encoded by ORF1 and ORF2 of Mucor hiemalis virus 1–4, the EMBOSS Needle pairwise alignment tool accessed via the website of the European Bioinformatics Institute (EMBL-EBI; https://www.ebi.ac.uk/Tools/msa/clustalo/ (accessed on 26 October 2021).) was used. The alignment is presented in Supplementary Table S1.

**Figure 1:**
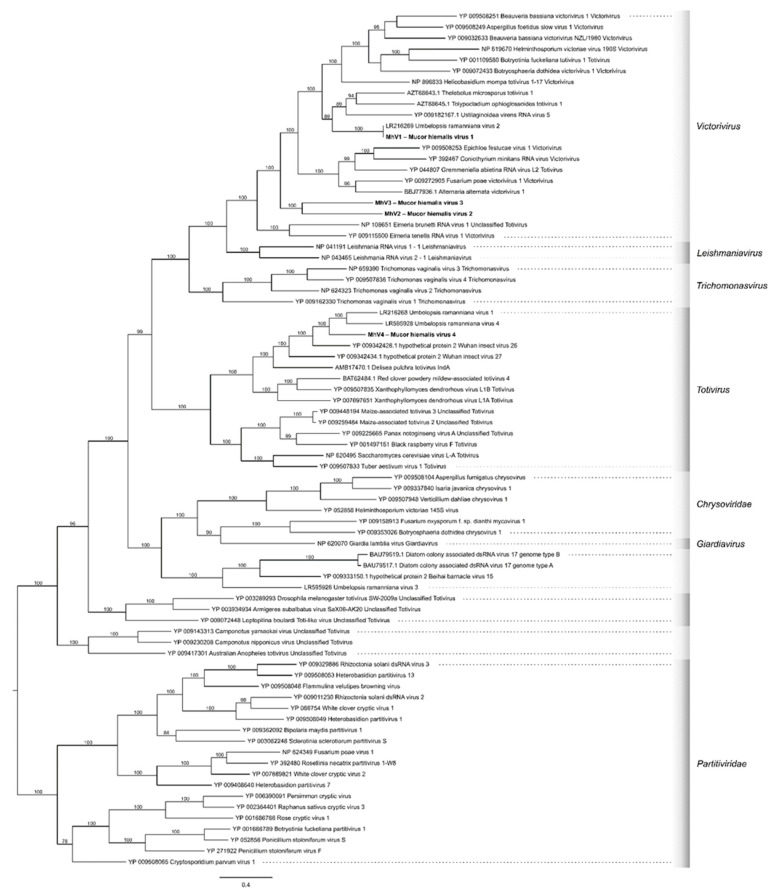
Phylogeny based on the RdRp amino acid sequences from representative members of the families *Totiviridae*, *Chrysoviridae,* and *Partitiviridae*. The resulting tree indicates that Mucor hiemalis virus 1 (MhV1), Mucor hiemalis virus 2 (MhV2), and Mucor hiemalis virus 3 (MhV3) are members of the genus *Victorivirus*, while Mucor hiemalis virus 4 (MhV4) belongs to the genus *Totivirus*. Maximum likelihood analysis was carried out by RAxML-NG v0.9.0 program [27], with 500 bootstrap replicates. The genetic distance was represented by the scale bar of 0.8 amino acid substitutions per nucleotide site. The novel *Mucor* mycoviruses are indicated with bold letters.

Representative RdRp sequences of *Totiviridae*, *Chrysoviridae,* and *Partitiviridae* were obtained from the viruSite (http://www.virusite.org/index.php (accessed on 26 October 2021).). The corresponding accession numbers are indicated on the tree (Figure 1). The dataset was supplemented by homologous hits of *U. ramanniana* RdRp sequences [7]. Multiple sequence alignment was carried out by PAGAN v.1.53 [23]. The best-fitting model for the phylogenetic inference was selected by using ModelTest-NG v0.1.4 [24], based on the Bayesian Information Criterion [25]. The selected model was LG [26] with four gamma categories. Maximum likelihood analysis was carried out by RAxML-NG v0.9.0 [27], with 500 bootstrap replicates.

### 2.7. Hybridization Studies

For hybridization, dsRNAs and control plasmids were separated by electrophoresis on 1.0% agarose/TAE (40 mM Tris/acetic acid, 1 mM EDTA, pH 7.6) horizontal gels. The relative sizes of the dsRNA molecules were estimated using DIG-labeled DNA Molecular Weight Marker VII (Roche, Mannheim, Germany) as size standards. To denature the dsRNA molecules, gel slides were rinsed in 0.05 M NaOH and 0.15 M NaCl buffer for 30 min and neutralized in 1 M Tris-HCl and 1.5 M NaCl buffer (pH 7.5) for 2 × 20 min as described by Hong et al. (1998) [28]. DNA samples (i.e., the control plasmids) were denatured in 0.5 M NaOH and 1.5 M NaCl buffer and neutralized in 0.5 M Tris and 1.5 M NaCl buffer (pH 7.5) [29]. Gel slides were blotted onto a positively charged nylon membrane (Amersham Hybond-N+, GE Healthcare) with 2× SSC buffer. Samples were allowed to dry at room temperature and immobilized with UV-crosslinking. Blots were hybridized using the DIG-labelled MhV1-4 CP and RdRp oligonucleotide probes in hybridization buffer (0.9 M NaCl, 1% SDS, 10% dextran sulfate) containing 5 µg/mL salmon sperm DNA (Invitrogen, Carlsbad, CA, USA). Probes were prepared by PCR from DNA templates in the presence of digoxigenin-UTP (DIG DNA Labeling Mix, Roche, Mannheim, Germany) using DreamTaq polymerase (Thermo Scientific, Vilnius, Lithuania). Primers used to amplify the probes are listed in the Supplementary Table S2. Hybridization was followed by immunological detection using alkaline phosphatase-conjugated anti-digoxigenin antibody (Roche, Mannheim, Germany). Reactions for detection were carried out according to the manufacturer’s instructions (Roche, Mannheim, Germany).

## 3. Results

### 3.1. Presence of dsRNA Elements in the Tested Mucor Hiemalis Strains

**Figure 2:**
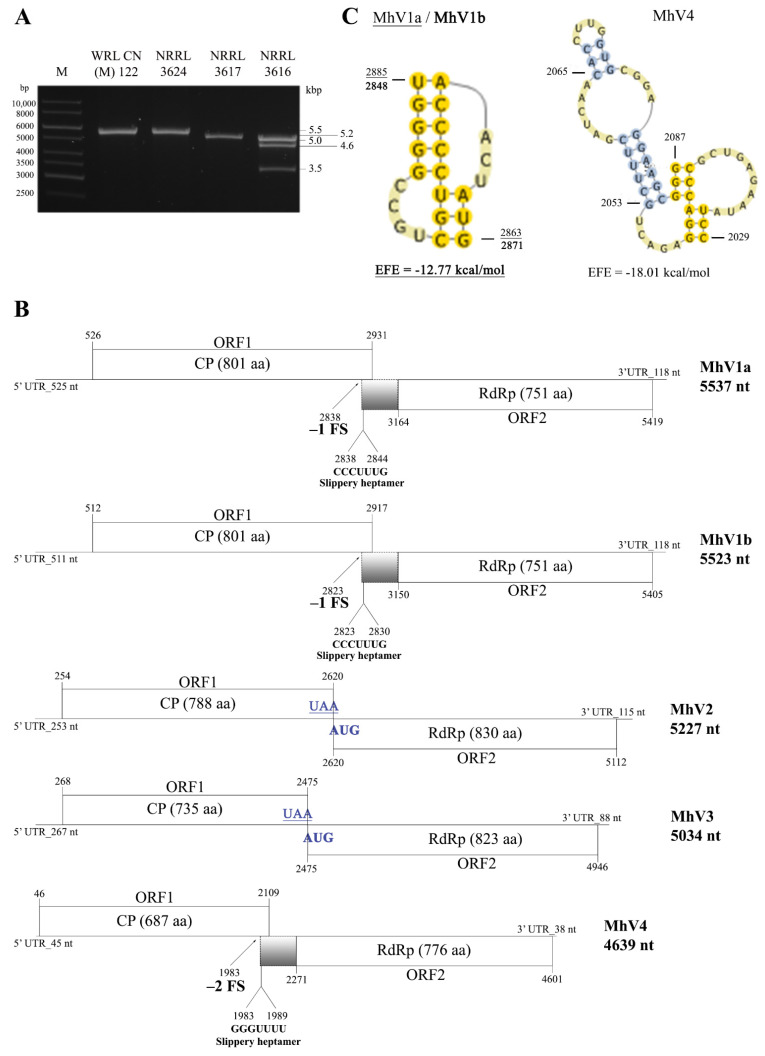
Gel electrophoresis pattern and genomic organization of the detected dsRNA genomes in four mycovirus-harboring *Mucor* strains. The complete gel can be found in the Supplementary material (Supplementary Figure S1) (**A**) Agarose gel electrophoresis of dsRNA fragments purified from the mycovirus-harboring *Mucor* strains. Lane M, GeneRuler 1 kb DNA Ladder (Thermo Scientific); Lane 1, *Mucor hiemalis* f. *hiemalis* WRL CN(M) 122; Lane 2, *Mucor hiemalis* f. *hiemalis* NRRL 3624; Lane 3, *Mucor hiemalis* f. *corticola* NRRL 3617; Lane 4, *Mucor hiemalis* f. *corticola* NRRL 3616. The sizes (kbp) of the detected dsRNA molecules and the corresponding virus genomes are indicated as well. (**B**) Genomic organization of the detected dsRNA genomes in *M. hiemalis* f. *hiemalis* WRL CN(M) 122, *M. hiemalis* f. *hiemalis* NRRL 3624, *M. hiemalis* f. *corticola* NRRL 3617, and *M. hiemalis* f. *corticola* NRRL 3616 strains, respectively, showing putative open reading frames (ORFs). The gray boxes indicate the possible beginning of the fusion protein by the corresponding frameshifting and the spacer region. Position and sequence of the potential slippery site is also marked in MhV1a, MhV1b, and MhV4. The stop-start codons (in blue color) indicate the possible ribosomal termination-reinitiation of MhV2 and of MhV3. Abbreviations: CP, coat protein; RdRp, RNA-dependent RNA polymerase. MhV1a, Mucor hiemalis virus 1a; MhV1b, Mucor hiemalis virus 1a; MhV2, Mucor hiemalis virus 2; MhV3, Mucor hiemalis virus 3; MhV4, Mucor hiemalis virus 4. (**C**) The pseudoknot structure downstream of the putative frameshift site of MhV1a in *M. hiemalis* f. *hiemalis* WRL CN(M) 122 (underline letters and numbers), MhV1b in *M. hiemalis* f. *hiemalis* NRRL 3624 (bold letters and numbers), and MhV4 in *M. hiemalis* f. *corticola* NRRL 3616 strains. The RNA H-type pseudoknots were predicted by the DotKnot program [21] and drawn by the PseudoViewer program [22]. EFE (kcal/mol) indicates the estimated free energy.

DsRNA molecules were detected in all four *M. hiemalis* strains tested (Table 1 and Figure 2A). In the four dsRNA-containing *Mucor* isolates, three different patterns were observed, with one or three discrete bands and estimated sizes ranging from 3.5 to 5.5 kbp (Table 1 and Figure 2A). 

### 3.2. Sequence and Genome Organization of the Detected dsRNA Elements

In the extracts of both tested *M. hiemalis* f. *hiemalis* strains, a single dsRNA molecule could be detected by gel electrophoresis with a 5.5-kbp estimated size **(**Figure 2A). By whole-genome sequence analysis, the dsRNAs were determined to be 5537 nt and 5523 nt in length for WRL CN(M) 122 and NRRL 3624, respectively. The two nucleotide sequences proved to be identical in 99%. Both dsRNAs contain two overlapped ORFs encoding the coat protein (CP) and the RNA-dependent RNA polymerase (RdRp). In the case of WRL CN(M) 122, untranslated regions (UTRs) of 525 and 118 nt were detected at the 5′ and 3′ termini, respectively, while UTRs of 511 and 118 nt were detected at the 5′ and 3′ termini of the genome found in NRRL 3624, respectively. For both genomes, predicted amino acid sequences of the CP and RdRp proteins show 99.8% and 100% identity, respectively. Based on these comparison results, it was deduced that both *M. hiemalis* f. *hiemalis* WRL CN(M) 122 and NRRL 3624 contain genome variants of the same virus species, which was tentatively named as Mucor hiemalis virus 1 (MhV1). The genome organization of the two variants (i.e., MhV1a and MhV1b for WRL CN(M) 122 and NRRL 3624, respectively) is presented in Figure 2B. For both genomes, ORF1 and ORF2 were predicted to be translated as a fusion protein via a –1 ribosomal frameshift. The same possible slippery heptamer and an H-type pseudoknot facilitating the programmed ribosomal frameshifting could be identified in both sequences (Figure 2C).

ORF1 (EMBL accession numbers are HG993402 and OU374593 for MhV1a and MhV1b, respectively) encodes a putative, 801-aa CP, while ORF2 (EMBL accession numbers are HG993403 and OU374594 for MhV1a and MhV1b, respectively) predicted to encode a putative, 751-aa RdRp. The predicted molecular weights of the proteins were found to be 83.97 and 81.76 kDa for the CP and the RdRp, respectively. BLASTp homology search with the corresponding sequences in the NCBI GenBank revealed a high degree of identity with CP and RdRp of viruses in the *Totiviridae* family; best matches are presented in Supplementary Table S3. The highest similarity was found to the Thelebolus microsporus totivirus 1 for CP and Umbelopsis ramanniana virus 2 for RdRp. We suggest that this dsRNA segment corresponds to a genomic component of a mycovirus in the *Totiviridae* family.

The *M. hiemalis* f. *corticola* NRRL 3617 nucleic acid extracts contained a dsRNA molecule with a size of 5227 nt determined by whole-genome sequence analysis (Figure 2A). The discovered dsRNA genome contains two ORFs in different frames (Figure 2B). ORF1 (from 254 to 2620 nt; EMBL accession number: HG993404) encodes a putative, 788-aa CP with an 82.65-kDa predicted molecular weight, while ORF2 (from 2620 to 5112 nt; EMBL accession number: HG993405) encodes a putative, 830-aa RdRp with a 91.4-kDa predicted molecular weight. UTRs of 253 and 115 nt were detected at the 5′ and 3′ termini, respectively. The stop (underlined) and start (bold) codons of ORF1 and ORF2, respectively, were found to be overlapped, forming an UA**A****UG** pentanucleotide (nt 2618 to 2622; Figure 2B). Such pentanucleotides are thought to be motifs for coupled translation termination and reinitialization [30,31]. BLASTp homology search with the corresponding sequences in the NCBI GenBank revealed a high degree of identity with the CP and RdRp of viruses in the *Totiviridae* family; best matches are presented in Supplementary Table S3. Based on these results, we suggest that this dsRNA corresponds to a genomic component of a novel mycovirus in the *Totiviridae* family, and we tentatively named it as Mucor hiemalis virus 2 (MhV2).

The fourth isolate, *M. hiemalis* f. *corticola* NRRL 3616, harbors three dsRNA molecules, with estimated sizes of 5.0, 4.6, and 3.5 kbp as detected by gel electrophoresis (Figure 2A). Among them, the largest molecule has a size of 5034 nt determined by whole-genome sequencing. It contains two ORFs in different frames. ORF1 was predicted to encode a 735-aa putative CP (from 268 to 2475 nt; EMBL accession number: HG993406), whereas ORF2 encodes an 823-aa putative RdRp (from 2475 to 4946 nt; EMBL accession number: HG993407). UTRs of 267 and 88 nt were detected at the 5′ and 3′ termini, respectively (Figure 2B). Similarly, to MhV2, the stop and start codons of ORF1 and ORF2, respectively, are overlapped, forming an UA**A****UG** pentanucleotide (nt 2473 to 2477; Figure 2B). The predicted molecular weights of the encoded proteins were found to be 77.92 and 91.69 kDa for the CP and the RdRp, respectively. BLASTp homology search with the corresponding sequences in the NCBI GenBank revealed a high degree of identity with the CP and RdRp of viruses in the *Totiviridae* family. Details of the five best matches are presented in Supplementary Table S3. We suggest that this dsRNA corresponds to a genomic component of a novel mycovirus in the *Totiviridae* family tentatively named as Mucor hiemalis virus 3 (MhV3). 

The second dsRNA in this strain is a 4639-nt molecule containing two overlapping ORFs (Figure 2A,B). ORF1 (from 46 to 2109 nt; EMBL accession number: HG993408) encodes a 687-nt putative CP with a 77.75-kDa predicted molecular weight; ORF2 (from 2271 to 4601; EMBL accession number: HG993409) was predicted to encode a putative, 776-aa RdRp protein with an 88.46-kDa predicted molecular weight. UTRs of 45 and 38 nt were detected at the 5′ and 3′ termini, respectively. ORF1 and ORF2 were predicted to be translated as a fusion protein via a rare +1 (or –2) ribosomal frameshift. A possible slippery heptamer and an H-type pseudoknot facilitating the programmed ribosomal frameshifting could be identified in the overlapping region (Figure 2C). Both encoded proteins showed the highest degree of identity with the Trichoderma koningiopsis totivirus 1 (Supplementary Table S3). This with another four matches given by the BLASTp query (Supplementary Table S3) indicates a high similarity to members of the *Totiviridae* family. As this sequence may correspond to a genomic dsRNA for a novel mycovirus, it was named as Mucor hiemalis virus 4 (MhV4).

A third, approximately 3.5-kb sized dsRNA fragment was detected by gel electrophoresis in the *M. hiemalis* f. *corticola* NRRL 3616 strain (Figure 2A). NGS sequencing also determined a 3383-nt sequence in the dsRNA extract of this fungus (NCBI accession no.: OK157910). The sequence contains a single ORF (from 28 to 3039 nt) and was predicted to encode a putative, 1003-aa hypothetical protein. Unfortunately, nucleic acid and the predicted amino acid sequence of this fragment did not show any similarities to known mycoviral sequences and other sequences in the GenBank by Blast queries.

### 3.3. Phylogenetic Analysis of the M. hiemalis Viruses 

Phylogeny was inferred by the ML method based on amino acid sequences of representative RdRp proteins from the *Totiviridae*, *Chrysoviridae,* and *Partitiviridae* families (Figure 1 and Supplementary Figure S2). In this phylogeny, MhV1a, MhV2, and MhV3 are part of the clade representing the genus *Victorivirus*, while MhV4 is seated in the clade containing viruses from the genus *Totivirus*. Within the *Victorivirus* clade, MhV2 and MhV3 form a new subclade and a sister group of other victoriviruses, such as Fusarium poae victorivirus 1. However, the EMBOSS Needle pairwise alignment tool (https://www.ebi.ac.uk/Tools/psa/emboss_needle/ (accessed on 26 October 2021).) found 30.3% and 33.6% sequence identity for the CP and RdRp proteins of MhV2 and MhV3, respectively, as it shown in Supplementary Table S1. At the same time, MhV1a/b proved to be very closely related to the recently discovered partial RdRp sequence named as Umbelopsis ramanniana virus 2 (UrV2) [7]. Within the *Totivirus* clade, MhV4 was found to be a sister group of Umbelopsis ramanniana virus 1 and 4. 

### 3.4. Detection of Virus Particles in M. hiemalis Strains

**Figure 3:**
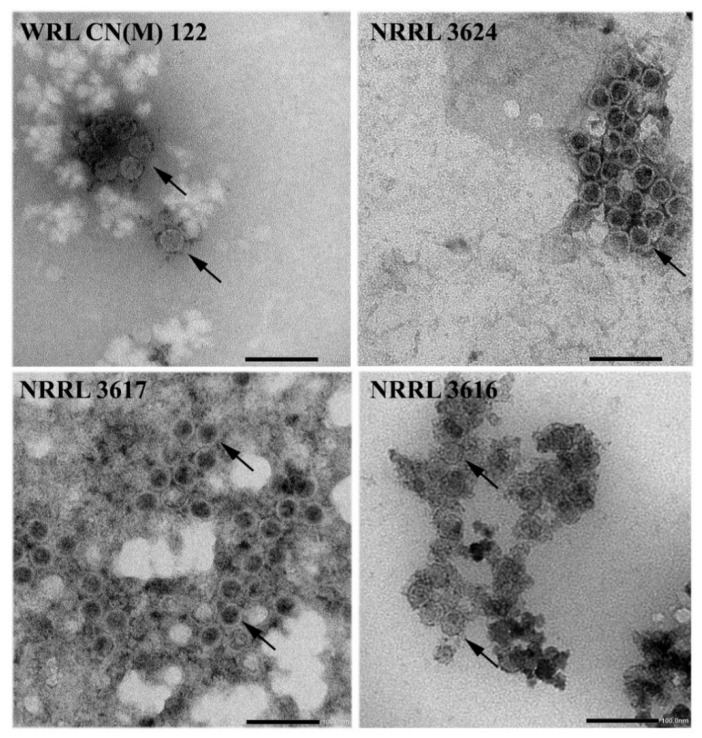
Morphology of virus particles detected in mycovirus-harboring Mucor strains. The virus particles were recovered by ultracentrifugation at 78,000× *g* for 12 h at 4 °C, which follows with sucrose gradient density centrifugation. Purified virus particles were negatively stained with 2% uranyl acetate in 50% ethanol for 5 min (3 times) and examined under a JEM-1400 Flash transmission electron microscope. VLPs about 33–36 nm in diameter were detected in the examined Mucor strains. The detected VLPs are indicated by black arrows.

Presence of isometric virus particles in the purified extracts of each Mucor hiemalis strain was confirmed by transmission electron microscopy. VLPs about 35 nm in diameter were detected in the MhV1a- and MhV1b-infected M. hiemalis f. hiemalis WRL CN(M)122 and NRRL 3624 strains. M. hiemalis f. corticola NRRL 3617 strain contained VLPs of 33 nm in diameter, while in the MhV3- and MhV4-infected M. hiemalis f. corticola NRRL 3616, size of the detected VLPs were found from 33 to 36 nm in diameter (Figure 3).

### 3.5. Hybridization Analysis of the dsRNA Patterns

**Figure 4:**
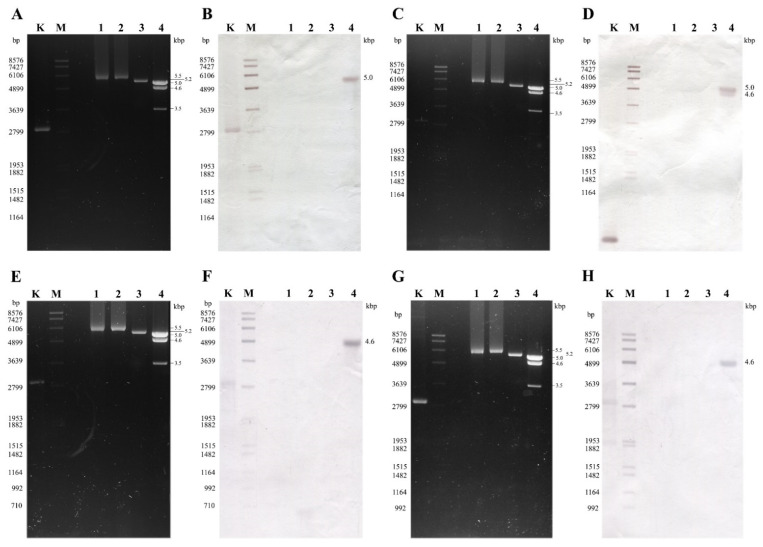
Northern blot analysis of dsRNA molecules purified from the mycovirus-harboring *Mucor* strains with probes designed using the MhV3 and MhV4 sequences. Panels (**A**,**C**,**E**,**G**): agarose gel electrophoresis of the dsRNA molecules purified from *M. hiemalis* f. *hiemalis* WRL CN(M) 122, *M. hiemalis* f. *hiemalis* NRRL 3624, *M. hiemalis* f. *corticola* NRRL 3617, and *M. hiemalis* f. *corticola* NRRL 3616. Lane K, control plasmid containing the corresponding PCR amplicon of virus genomes; Lane M, DIG-labeled DNA Molecular Weight Marker VII (Roche); Lane 1, *M. hiemalis* f. *hiemalis* WRL CN(M) 122; Lane 2, *M. hiemalis* f. *hiemalis* NRRL 3624; Lane 3, *M. hiemalis* f. *corticola* NRRL 3617; Lane 4, *M. hiemalis* f. *corticola* NRRL 3616. Right numbers indicate the sizes (kbp) of the detected dsRNA molecules. Panels (**B**,**D**): Northern blot analysis of the dsRNA molecules extracted from *M. hiemalis* f. *corticola* NRRL 3616 using the MhV3 CP and MhV3 RdRp probes, respectively. In panel (**B**), MhV3 CP probe gave strong hybridization signal to the largest, 5.0-kbp band and the *Bgl*II digested control plasmid, which contains the PCR amplicon of the MhV3 CP probe sequence. In panel (**D**), MhV3 RdRp probe gave strong hybridization signal to the largest, 5.0-kbp band but also hybridized to the 4.6-kbp band. The *Bgl*II digested control plasmid containing the PCR amplicon of the MhV3 RdRp probe sequence gave a strong hybridization signal with the MhV3 RdRp probe. Panels (**F**,**H**), Northern blot analysis of the dsRNA molecules purified from *M. hiemalis* f. *corticola* NRRL 3616 using the MhV4 CP and MhV4 RdRp probes, respectively. In both (**F**,**H**) panels, the probes designed for MhV4 CP and MhV4 RdRp sequences hybridized to the second largest, 4.6-kbp band and the *Bgl*II digested control plasmid, which contains the PCR amplicon of the MhV4 CP and MhV4 RdRp probe sequences.

Probes designed for the CP and the RdRp genes of MhV1a hybridized to the 5.5 kbp dsRNA electrophoretic pattern of *M. hiemalis* f. *hiemalis* WRL CN(M) 122 and *M. hiemalis* f. *hiemalis* NRRL 3624 strains (Supplementary Figure S3A–D), indicating that both isolates carry the genome variants of the same virus species. The probe designed for the MhV2 CP and RdRp sequences gave signal to the 5.2-kb sized dsRNA bands of *M. hiemalis* f. *corticola* NRRL 3617 (Supplementary Figure S3E–H). The probe based on the CP sequence of the MhV3 molecule hybridized to the largest, 5.0-kbp dsRNA electrophoretic pattern of *M. hiemalis* f. *corticola* NRRL 3616 (Figure 4A,B), while the probe designed for the RdRp genes of MhV3 hybridized to the largest, 5.0-kbp and the 4.6-kbp dsRNA bands of the same pattern (Figure 4C,D). The CP and RdRp probes, designed for CP and RdRp genes of MhV4, both hybridized to the 4.6-kbp dsRNA molecule of *M. hiemalis* f. *corticola* strain (Figure 4E–H).

## 4. Discussion

Viruses of Mucorales fungi are still highly unexplored. Although there are some traits concerning the virus harboring of *M. hiemalis*, mycoviruses have not previously been described from this species. Earlier, Vágvölgyi et al. (1998) tested 123 Mucor isolates and detected similar dsRNA patterns in two *M. corticola* and one *M. hiemalis* strains, but the genome sequence and VLP harboring were not reported [13]. In this study, we examined two *M. hiemalis* f. *hiemalis* and two *M. hiemalis* f. *corticola* strains for the presence of dsRNA molecules, and the discovery of five independent viral genomes corresponding to four mycoviruses (i.e., MhV1–4) was proven.

The tested *M. hiemalis* f. *hiemalis* strains most probably contain members of the same virus species, MhV1, indicating that it was acquired by their common ancestor. Although the amino acid sequences of the CP and the RdRp proved to be identical, the two genomes display a certain level of difference in their nucleotide sequences, which corresponds to the independent history (Table 1) of these closely related fungi. 

The two *M. hiemalis* f. *corticola* strains harbor different viruses although MhV2 and MhV3 seem to be closely related within the *Victorivirus* genus. Moreover, the strain NRRL 3616 contain a further virus, MhV4, which belongs to the *Totivirus* genus, indicating that this strain harbor multiple mycoviruses. Mixed infections have also been reported from other fungi, such as from *Beauveria bassiana* [32] or the Mucoromycota fungus, *U. ramanniana* [7]. In a recent publication, nine mycoviruses belonging to eight potential families were identified in a single *Sclerotinia sclerotiorum* strain [33].

All discovered viruses belong to the Totiviridae family. Within the genus *Victorivirus*, MhV1 seems to be closely related to a partial genome recently described from *Umbelopsis ramanniana* (i.e., Umbelopsis ramanniana virus 2) [7]. Similarly, MhV4 was found to be closely related to the new totiviruses also discovered in *U. ramanniana* (i.e., UrV1 and UrV4) [7]. It seems that MhV1 and MhV4 have the closest relatives among mycoviruses infecting Mucoromycota fungi. Interestingly, the amino acid sequence of the CP and RdRp of MhV4 show 86 and 90% identity with the CP and RdRp sequences of Trichoderma koningiopsis totivirus 1 (TkTV1), respectively (see Supplementary Table S3), which suggest the common origin of these viruses. It is worth mentioning that *Trichoderma koningiopsis* is a known mycoparasite. Furthermore, Khalifa and MacDiarmid recently detected TkTV1 in a *T. koningiopsis* strain isolated from a sample, which also contained a *Clonostachys rosea* strain [34]. The latter fungus harbored a virus, of which the genome proved to be nearly identical with that of TkTV1, suggesting the possibility of a natural horizontal transmission event. Up to date, we do not have information that *Trichoderma* species mycoparasite on *Mucor* isolates, but we cannot exclude the possibility of viral horizontal transfer between them. 

Although sequence and phylogenetic analyses identified MhV1 as a member of the genus *Victorivirus*, genome structure of MhV1a and MhV1b was found to be different from that of the most victoriviruses. In this genus, RdRp is generally translated separately from the CP by a stop-reinitiation or a coupled termination-reinitiation mechanism [35,36]. Characteristic motifs of these reinitiation mechanisms are the “AUGA” and “UAAUG” tetra- and pentanucleotides, respectively, which correspond to the overlapping stop and start codons of ORF1 and ORF2, respectively [35,37,38,39,40]. Such motifs could not be found in the MhV1 genomes; instead, an overlapping region and motifs (i.e., a putative slippery site and an H-type pseudoknot) suggest the translation of a fusion protein via a programmed -1 ribosomal frameshift could be proposed (Figure 2). This genome organization is rather characteristic to the genus *Totivirus* [41,42]. A similar situation was found in case of the recently described Alternaria alternata victorivirus 1, which also proved to have a *Totivirus*-like genome organization [39]. Although RdRps of both viruses seated into the genus Victorivirus in our phylogeny inferred from the RdRps of dsRNA mycoviruses (Figure 1), they seem to be not closely related, as they belong to different subclades.

Both examined *M. hiemalis* f. *corticola* strains contained a closely related *Victorivirus* genome (i.e., MhV2 and MhV3), which show the organization characteristic to this genus with the abovementioned “UAAUG” pentanucleotide. This motif is thought to assist in the coupled termination-reinitiation mechanism during the translation of the two ORFs [31].

The MhV4 genome has a *Totivirus*-type organization, suggesting the translation of a fusion protein [39,41,42]. However, it was also suggested that it may be carried out via a programmed +1 (or −2) ribosomal frameshift (Figure 2), which is a rare mechanism in *Totivirus*. Within the *Totiviridae*, examples for +1 frameshifting were rather reported in the genera Trichomonas- and Leishmaniavirus [43,44].

VLPs were found in all four *Mucor* strains and the detected virus particles have a typical shape and about 30- to 40-nm particle size (Figure 3), which is characteristic to the *Totiviridae* family [6]. VLPs with the same size were found in *M. hiemalis* f. *hiemalis* WRL CN(M)122 and NRRL 3624 strains, which also support the closeness of MhV1a and MhV1b.

Due to identification and molecular characterization of novel viruses of Mucoromycota fungi, we can obtain insight into their mycoviral diversity as well as into the mycoviral phylogeny and genome organization. Since we identified certain unique characteristics, as it can be seen above, which were barely described previously, this points to the fact that our knowledge about viruses of Mucoromycota fungi is still insufficient. Therefore, it is necessary to investigate more thoroughly the so-called early-diverging lineages of fungi, including Mucoromycota. 

## Figures and Tables

**Table 1:** Sizes of dsRNA molecules detected in *Mucor* isolates.

Species Name	Collection Number	Other Collection Number	Origin/Other Information	dsRNA Size (kb)	Detected Viruses
*Mucor hiemalis* f. *hiemalis*	WRL CN(M) 122 ^a^	SZMC 12,034 ^b^	Oak-bark, UK	5.5	MhV1a ^e^
*Mucor hiemalis* f. *hiemalis*	NRRL 3624 ^c^	CBS 201.65 ^d^	USA, Type strain of *M. hiemalis*	5.5	MhV1b ^f^
*Mucor hiemalis* f. *corticola*	NRRL 3617	CBS 366.68	soil, Austria	5.2	MhV2 ^g^
*Mucor hiemalis* f. *corticola*	NRRL 3616	CBS 365.58	soil, Austria	5.0; 4.6; 3.5	MhV3 ^h^; MhV4 ^i^

Abbreviations: ^a^ WRL, Wellcome Bacterial Collection, Beckenham, UK; ^b^ SZMC, Szeged Microbiology Collection, Szeged, Hungary; ^c^ NRRL, Agricultural Research Service Culture Collection, Peoria, Illinois; ^d^ CBS, Westerdijk Fungal Biodiversity Institute, Utrecht, The Netherlands; ^e^ MhV1a, Mucor hiemalis virus 1a; ^f^ MhV1b, Mucor hiemalis virus 1a; ^g^ MhV2, Mucor hiemalis virus 2; ^h^ MhV3, Mucor hiemalis virus 3; ^i^ MhV4, Mucor hiemalis virus 4.

## Data Availability

All data generated or analysed during this study are included in this article and its supplementary information files. Strains used are available from the Szeged Microbiological Collection (www.szmc.hu, accessed on 27 September 2021; email: pappt@bio.u-szeged.hu).

## Supplementary Materials

The following are available online at https://www.mdpi.com/article/10.3390/v13112319/s1, Table S1: Sequence identities of proteins encoded by ORF1 and ORF2 of the virus genomes identified in *Mucor hiemalis* strains. Table S2: List of primers applying for amplification of cDNAs and hybridization probes. Table S3: Amino acid sequence identities of the MhV1a/b CP, MhV1a/b RdRp, MhV2 CP, MhV2 RdRp, MhV3 CP, MhV3 RdRp, MhV4 CP and MhV4 RdRp. Figure S1: Gel electrophoresis pattern of the detected dsRNA genomes in four mycovirus-harboring *Mucor* strains. Figure S2: Maximum likelihood phylogenetic tree with extended taxon sampling from the genera *Totivirus* and *Victorivirus*. Figure S3: Northern blot analysis of dsRNA molecules purified from the mycovirus-harboring *Mucor* strains with probes designed using the MhV1a and MhV2 sequences.
