# Supplementary material for: Characterization of Four Novel dsRNA Viruses Isolated from Mucor hiemalis Strains
Source: Viruses. 2021 Nov 21;13(11):2319. doi: 10.3390/v13112319 (PMC8625083; doi:10.3390/v13112319)
Supplement: Supplementary file 1 [file viruses-13-02319-s001.zip › Supplementary Figure S1.pdf]

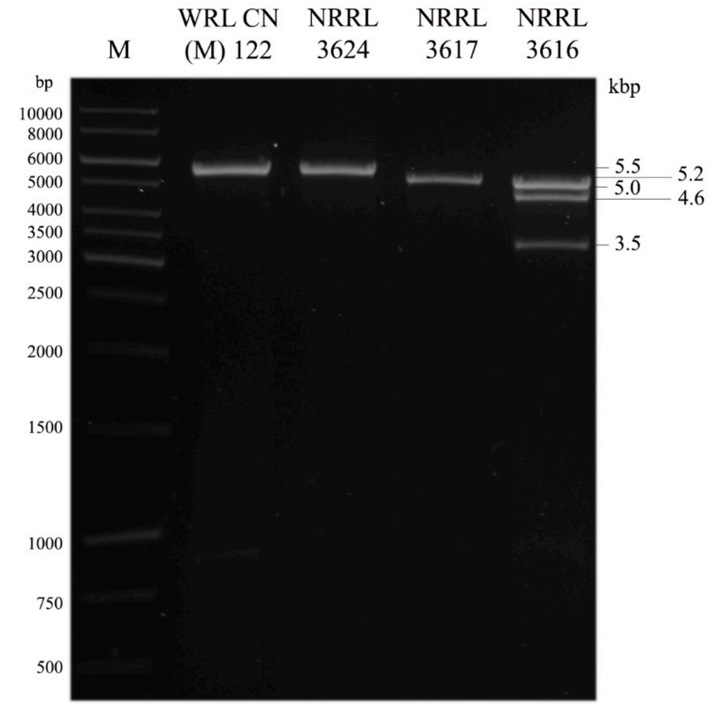

Supplementary Figure S1. Gel electrophoresis pattern of the detected dsRNA genomes in four mycovirus-harboring *Mucor* strains. Agarose gel electrophoresis of dsRNA fragments purified from the mycovirus-harboring *Mucor* strains. Lane M, GeneRuler 1 kb DNA Ladder (Thermo Scientific); Lane 1, *Mucor hiemalis* f. *hiemalis* WRL CN(M) 122; Lane 2, *Mucor hiemalis* f. *hiemalis* NRRL 3624; Lane 3, *Mucor hiemalis* f. *corticola* NRRL 3617; Lane 4, *Mucor hiemalis* f. *corticola* NRRL 3616. The sizes (kbp) of the detected dsRNA molecules and the corresponding virus genomes are indicated as well.
