# Supplementary material for: Characterization of Four Novel dsRNA Viruses Isolated from Mucor hiemalis Strains
Source: Viruses. 2021 Nov 21;13(11):2319. doi: 10.3390/v13112319 (PMC8625083; doi:10.3390/v13112319)
Supplement: Supplementary file 1 [file viruses-13-02319-s001.zip › Supplementary Figure S2.pdf]

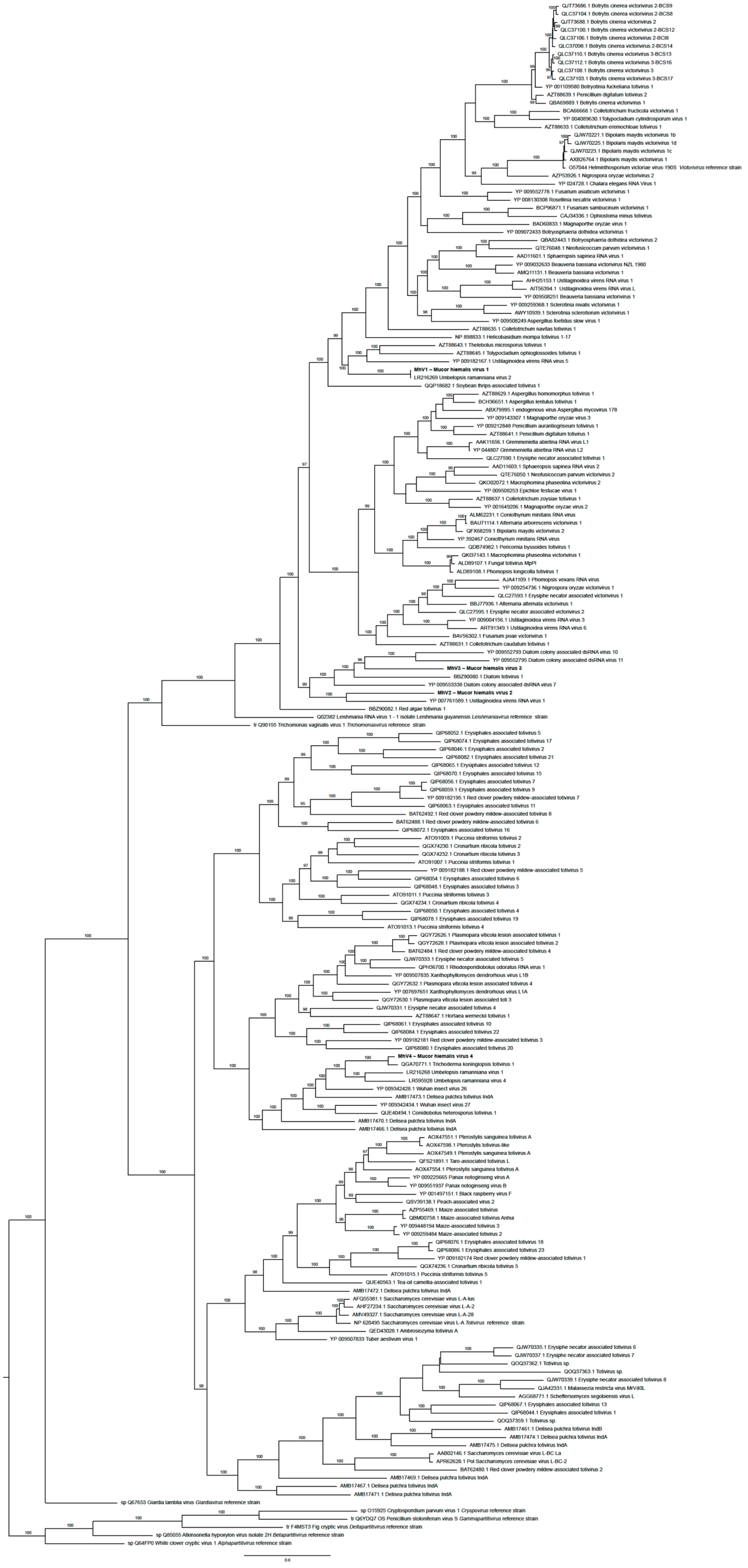
QJT73686.1 Botrytis cinerea victorivirus 2-BCS9
QLC37104.1 Botrytis cinerea victorivirus 2-BCS8
QJT73688.1 Botrytis cinerea victorivirus 2
QLC37100.1 Botrytis cinerea victorivirus 2-BCS12
QLC37106.1 Botrytis cinerea victorivirus 2-BCI8
QLC37098.1 Botrytis cinerea victorivirus 2-BCS14
QLC37110.1 Botrytis cinerea victorivirus 3-BCS13
QLC37112.1 Botrytis cinerea victorivirus 3-BCS16
QLC37108.1 Botrytis cinerea victorivirus 3
QLC37103.1 Botrytis cinerea victorivirus 3-BCS17
YP 001109580 Botryotinia fuckeliana totivirus 1
AZT88639.1 Penicillium digitatum totivirus 2
QBA69889.1 Botrytis cinerea victorivirus 1
BCA66668.1 Colletotrichum fructicola victorivirus 1
YP 004089630.1Tolypocladium cylindrosporum virus 1
AZT88633.1 Colletotrichum eremochloae totivirus 1
QJW70221.1 Bipolaris maydis victorivirus 1b
QJW70225.1 Bipolaris maydis victorivirus 1d
QJW70223.1 Bipolaris maydis victorivirus 1c
AXB26764.1 Bipolaris maydis victorivirus 1
O57044 Helminthosporium victoriae virus-190S *Victorivirus* reference strain
AZP53926.1 Nigrospora oryzae victorivirus 2
YP 024728.1 Chalara elegans RNA Virus 1
YP 009552778.1 Fusarium asiaticum victorivirus 1
YP 008130308 Rosellinia necatrix victorivirus 1
BCP96871.1 Fusarium sambucinum victorivirus 1
CAJ34336.1 Ophiostoma minus totivirus
BAD60833.1 Magnaporthe oryzae virus 1
YP 009072433 Botryosphaeria dothidea victorivirus 1
QBA82443.1 Botryosphaeria dothidea victorivirus 2
QTE76048.1 Neofusicoccum parvum victorivirus 1
AAD11601.1 Sphaeropsis sapinea RNA virus 1
YP 009032633 Beauveria bassiana victorivirus NZL 1980
AMQ11131.1 Beauveria bassiana victorivirus 1
AHH25153.1 Ustilaginoidea virens RNA virus 1
AIT56394.1 Ustilaginoidea virens RNA virus L
YP 009508251 Beauveria bassiana victorivirus 1
YP 009259368.1 Sclerotinia nivalis victorivirus 1
AWY10939.1 Sclerotinia sclerotiorum victorivirus 1
YP 009508249 Aspergillus foetidus slow virus 1
AZT88635.1 Colletotrichum navitas totivirus 1
NP 898833.1 Helicobasidium mompa totivirus 1-17
AZT88643.1 Thelebolus microsporus totivirus 1
AZT88645.1 Tolypocladium ophioglossoides totivirus 1
YP 009182167.1 Ustilaginoidea virens RNA virus 5
MhV1 – Mucor hiemalis virus 1
LR216269 Umbelopsis ramanniana virus 2
QQP18682.1 Soybean thrips-associated totivirus 1
AZT88629.1 Aspergillus homomorphus totivirus 1
BCH36651.1 Aspergillus lentulus totivirus 1
ABX79995.1 endogenous virus Aspergillus mycovirus 178
YP 009143307.1 Magnaporthe oryzae virus 3
YP 009212848 Penicillium aurantiogriseum totivirus 1
AZT88641.1 Penicillium digitatum totivirus 1
AAK11656.1 Gremmeniella abietina RNA virus L1
YP 044807 Gremmeniella abietina RNA virus L2
QLC27590.1 Erysiphe necator associated totivirus 1
AAD11603.1 Sphaeropsis sapinea RNA virus 2
QTE76050.1 Neofusicoccum parvum victorivirus 2
QKO02072.1 Macrophomina phaseolina victorivirus 2
YP 009508253 Epichloe festucae virus 1
AZT88637.1 Colletotrichum zoysiae totivirus 1
YP 001649206.1 Magnaporthe oryzae virus 2
ALM62231.1 Coniothyrium minitans RNA virus
BAU71114.1 Alternaria arborescens victorivirus 1
QFX68259.1 Bipolaris maydis victorivirus 2
YP 392467 Coniothyrium minitans RNA virus
QDB74982.1 Pericornia byssoides totivirus 1
QKI37143.1 Macrophomina phaseolina victorivirus 1
ALD89107.1 Fungal totivirus MpPI
ALD89108.1 Phomopsis longicolla totivirus 1
AJA41109.1 Phomopsis vexans RNA virus
YP 009254736.1 Nigrospora oryzae victorivirus 1
QLC27593.1 Erysiphe necator associated victorivirus 1
BBJ77936.1 Alternaria alternata victorivirus 1
QLC27595.1 Erysiphe necator associated victorivirus 2
YP 009004156.1 Ustilaginoidea virens RNA virus 3
ART91349.1 Ustilaginoidea virens RNA virus 6
BAV56302.1 Fusarium poae victorivirus 1
AZT88631.1 Colletotrichum caudatum totivirus 1
YP 009552793 Diatom colony associated dsRNA virus 10
YP 009552795 Diatom colony associated dsRNA virus 11
MhV3 – Mucor hiemalis virus 3
BBZ90080.1 Diatom totivirus 1
YP 009553338 Diatom colony associated dsRNA virus 7
MhV2 – Mucor hiemalis virus 2
YP 007761589.1 Ustilaginoidea virens RNA virus 1
BBZ90082.1 Red algae totivirus 1
Q02382 Leishmania RNA virus 1 - 1 isolate Leishmania guyanensis *Leishmaniavirus* reference strain
tr Q90155 Trichomonas vaginalis virus 1 *Trichomonasvirus* reference strain
QIP68052.1 Erysiphales associated totivirus 5
QIP68074.1 Erysiphales associated totivirus 17
QIP68046.1 Erysiphales associated totivirus 2
QIP68082.1 Erysiphales associated totivirus 21
QIP68065.1 Erysiphales associated totivirus 12
QIP68070.1 Erysiphales associated totivirus 15
QIP68056.1 Erysiphales associated totivirus 7
QIP68059.1 Erysiphales associated totivirus 9
YP 009182195.1 Red clover powdery mildew-associated totivirus 7
QIP68063.1 Erysiphales associated totivirus 11
BAT62492.1 Red clover powdery mildew-associated totivirus 8
BAT62488.1 Red clover powdery mildew-associated totivirus 6
QIP68072.1 Erysiphales associated totivirus 16
ATO91009.1 Puccinia striiformis totivirus 2
QGX74230.1 Cronartium ribicola totivirus 2
QGX74232.1 Cronartium ribicola totivirus 3
ATO91007.1 Puccinia striiformis totivirus 1
YP 009182188.1 Red clover powdery mildew-associated totivirus 5
QIP68054.1 Erysiphales associated totivirus 6
QIP68048.1 Erysiphales associated totivirus 3
ATO91011.1 Puccinia striiformis totivirus 3
QGX74234.1 Cronartium ribicola totivirus 4
QIP68050.1 Erysiphales associated totivirus 4
QIP68078.1 Erysiphales associated totivirus 19
ATO91013.1 Puccinia striiformis totivirus 4
QGY72626.1 Plasmopara viticola lesion associated totivirus 1
QGY72628.1 Plasmopara viticola lesion associated totivirus 2
BAT62484.1 Red clover powdery mildew-associated totivirus 4
QJW70333.1 Erysiphe necator associated totivirus 5
QPH36700.1 Rhodosporidiobolus odoratus RNA virus 1
YP 009507835 Xanthophyllomyces dendrorhous virus L1B
QGY72632.1 Plasmopara viticola lesion associated totivirus 4
YP 007697651 Xanthophyllomyces dendrorhous virus L1A
QGY72630.1 Plasmopara viticola lesion associated toti 3
QJW70331.1 Erysiphe necator associated totivirus 4
AZT88647.1 Hortaea werneckii totivirus 1
QIP68061.1 Erysiphales associated totivirus 10
QIP68084.1 Erysiphales associated totivirus 22
YP 009182181 Red clover powdery mildew-associated totivirus 3
QIP68080.1 Erysiphales associated totivirus 20
MhV4 – Mucor hiemalis virus 4
QGA70771.1 Trichoderma koningiopsis totivirus 1
LR216268 Umbelopsis ramanniana virus 1
LR595928 Umbelopsis ramanniana virus 4
YP 009342428.1 Wuhan insect virus 26
AMB17473.1 Delisea pulchra totivirus IndA
YP 009342434.1 Wuhan insect virus 27
QUE40494.1 Conidiobolus heterosporus totivirus 1
AMB17470.1 Delisea pulchra totivirus IndA
AMB17466.1 Delisea pulchra totivirus IndA
AOX47551.1 Pterostylis sanguinea totivirus A
AOX47598.1 Pterostylis totivirus-like
AOX47549.1 Pterostylis sanguinea totivirus A
QFS21891.1 Taro-associated totivirus L
AOX47554.1 Pterostylis sanguinea totivirus A
YP 009225665 Panax notoginseng virus A
YP 009551937 Panax notoginseng virus B
YP 001497151.1 Black raspberry virus F
QSV39138.1 Peach-associated virus 2
AZP55469.1 Maize associated totivirus
QBM00758.1 Maize-associated totivirus Anhui
YP 009448194 Maize-associated totivirus 3
YP 009259484 Maize-associated totivirus 2
QIP68076.1 Erysiphales associated totivirus 18
QIP68086.1 Erysiphales associated totivirus 23
YP 009182174 Red clover powdery mildew-associated totivirus 1
QGX74236.1 Cronartium ribicola totivirus 5
ATO91015.1 Puccinia striiformis totivirus 5
QUE40563.1 Tea-oil camellia-associated totivirus 1
AMB17472.1 Delisea pulchra totivirus IndA
AFQ55381.1 Saccharomyces cerevisiae virus L-A-lus
AHF27234.1 Saccharomyces cerevisiae virus L-A-2
AMV49327.1 Saccharomyces cerevisiae virus L-A-28
NP 620495 Saccharomyces cerevisiae virus L-A *Totivirus* reference strain
QED43028.1 Ambrosiozyma totivirus A
YP 009507833 Tuber aestivum virus 1
QJW70335.1 Erysiphe necator associated totivirus 6
QJW70337.1 Erysiphe necator associated totivirus 7
QOQ37362.1 Totivirus sp.
QOQ37363.1 Totivirus sp.
QJW70339.1 Erysiphe necator associated totivirus 8
QJA42331.1 Malassezia restricta virus MrV40L
AGG68771.1 Scheffersomyces segobiensis virus L
QIP68067.1 Erysiphales associated totivirus 13
QIP68044.1 Erysiphales associated totivirus 1
QOQ37359.1 Totivirus sp.
AMB17461.1 Delisea pulchra totivirus IndB
AMB17474.1 Delisea pulchra totivirus IndA
AMB17475.1 Delisea pulchra totivirus IndA
AAB02146.1 Saccharomyces cerevisiae virus L-BC La
APR62628.1 Pol Saccharomyces cerevisiae virus L-BC-2
BAT62480.1 Red clover powdery mildew-associated totivirus 2
AMB17469.1 Delisea pulchra totivirus IndA
AMB17467.1 Delisea pulchra totivirus IndA
AMB17471.1 Delisea pulchra totivirus IndA
sp Q67653 Giardia lamblia virus *Giardiavirus* reference strain
sp O15925 Cryptosporidium parvum virus 1 *Cryspovirus* reference strain
tr Q6YDQ7 OS Penicillium stoloniferum virus S *Gammapartitivirus* reference strain
tr F4MST3 Fig cryptic virus *Deltapartitivirus* reference strain
sp Q85055 Atkinsonella hypoxylon virus isolate 2H *Betapartitivirus* reference strain
sp Q64FP0 White clover cryptic virus 1 *Alphapartitivirus* reference strain
0.6

Supplementary Figure S2: Maximum likelihood phylogenetic tree with extended taxon sampling from the genera *Totivirus* and *Victorivirus*. Multiple sequence alignment was done by PAGAN v.1.53 and the Phylogenetic inference was carried out by using IQ-TREE v.1.6.1 with the LG+G4 evolutionary model. Statistical support of branches was calculated with 5000 UFBoot replicates. Only values higher than 95% are shown.
