# Supplementary material for: Characterization of Four Novel dsRNA Viruses Isolated from Mucor hiemalis Strains
Source: Viruses. 2021 Nov 21;13(11):2319. doi: 10.3390/v13112319 (PMC8625083; doi:10.3390/v13112319)
Supplement: Supplementary file 1 [file viruses-13-02319-s001.zip › Supplementary Table S1.pdf]

Supplementary Table S1 Sequence identities of proteins encoded by ORF1 and ORF2 of the virus genomes identified in *M. hiemalis* f. *hiemalis* WRL CN(M)12, *Mucor hiemalis* f. *hiemalis* NRRL 3624 *M. hiemalis* f. *corticola* NRRL 3617, *M. hiemalis* f. *corticola* NRRL 3616.

**Virus genomes**	**sequence identity (%)**	
	**ORF1**	**ORF2**
MhV1a – MhV1b	99.8	100.0
MhV1a – MhV2	27.9	34.2
MhV1a – MhV3	31.4	34.7
MhV1a – MhV4	17.1	19.8
MhV2 – MhV3	30.3	33.6
MhV2 – MhV4	15.7	21.9
MhV3 – MhV4	13.1	20.7

MhV1a: Mucor hiemalis virus 1a; MhV1b: Mucor hiemalis virus 1b; MhV2: Mucor hiemalis virus 2; MhV3: Mucor hiemalis virus 3; MhV4: Mucor hiemalis virus 4; ORF1: coat protein; ORF2: RNA dependent RNA polymerase.
