# Supplementary material for: Characterization of Four Novel dsRNA Viruses Isolated from Mucor hiemalis Strains
Source: Viruses. 2021 Nov 21;13(11):2319. doi: 10.3390/v13112319 (PMC8625083; doi:10.3390/v13112319)
Supplement: Supplementary file 1 [file viruses-13-02319-s001.zip › Supplementary Table S2.pdf]

Supplementary Table S2 List of primers applying for amplification of cDNAs and hybridization probes.

Primer*	Sequence (5′ - 3′)	Application	Reference
PC3-T7 loop	p-GGATCCCGGGAATTCGGTAATACGACT CACTATATTTTTATAGTGAGTCGTATTA-OH	self-priming anchor primer for cDNA synthesis	Potgieter et al., 2009
PC2	p–CCGAATTCCCGGGATCC	Amplification of cDNA	Potgieter et al., 2009
MhV1CP F	CACTCTACCCTTCAACTTCAACC	Amplification of probe for MhV1 CP	In this study
MhV1CP R	TAGACACCGATGTTGTAGTGGA		
MhV1RdRp F	ACCCGTTCCATCTTCCCT	Amplification of probe for MhV1 RdRp	In this study
MhV1RdRp R	TGTTTCCAGCAAGGTCGG		
MhV2CP F	CCTACCGAACCACCGAAATGTC	Amplification of probe for MhV2 CP	In this study
MhV2CP R	CTGATAAGATCCCTGCCACGA		
MhV2RdRp F	TTTGGACTTCACGCTGGTTATGG	Amplification of probe for MhV2 RdRp	In this study
MhV2RdRp R	GCCCTTTCCAGACCTCTTCG		
MhV3CP F	ACACTCGTTTGCGTTCTTATTCTG	Amplification of probe for MhV3 CP	In this study
MhV3CP R	CCTCACTGACACCAGAAATGC		
MhV3RdRp F	GTGGTGTTGGTGAGATTGATTTGC	Amplification of probe for MhV3 RdRp	In this study
MhV3RdRp R	CGTTCAACCATGCCAACAAGAC		
MhV4CP F	ACACTCTGATACATAACGAAGCCA	Amplification of probe for MhV4 CP	In this study
MhV4CP R	ATCGTTGACCATAGTTCCCAAGAC		
MhV4RdRp F	TTTGCCTAAGCTACCGTTCAACC	Amplification of probe for MhV4 RdRp	In this study
MhV4RdRp R	AACATAAGTAGTCAGTCTCCAGCC		

* F and R indicating forward and reverse primers, respectively.

MhV1a/b_KN_R5	GGAAATCCTCGGAACCCTCC	Amplification of 5′ terminus of MhV1a/b	In this study
MhV1a/b_KN_F3	GCCTACCCTCGCTTCAAAGC	Amplification of 3′ terminus of MhV1a/b	In this study
MhV2_KN_R5	GCTCGCATAGCTGGGTTCAG	Amplification of 5′ terminus of MhV2	In this study
MhV2_KN_F3	GGTCATGGTAGAGTCTTCATTCTC	Amplification of 3′ terminus of MhV2	In this study
MhV3_KN_R5	ACTCACTTGAATTGAACAGTCACC	Amplification of 5′ terminus of MhV3	In this study
MhV3_KN_F3	TATGTCACCTGTTATTTCTGCTCG	Amplification of 3′ terminus of MhV3	In this study
MhV4_KN_R5	GTGCCTGTGTATAAAGCCGA	Amplification of 5′ terminus of MhV4	In this study
MhV4_KN_F3	TGGTCAAAGAGAAAGGTCAG	Amplification of 3′ terminus of MhV4	In this study
