# Supplementary material for: Characterization of Four Novel dsRNA Viruses Isolated from Mucor hiemalis Strains
Source: Viruses. 2021 Nov 21;13(11):2319. doi: 10.3390/v13112319 (PMC8625083; doi:10.3390/v13112319)
Supplement: Supplementary file 1 [file viruses-13-02319-s001.zip › Supplementary Table S3.pdf]

Supplementary Table S3. Amino acid sequence identities of the MhV1a/b CP, MhV1a/b RdRp, MhV2 CP, MhV2 RdRp, MhV3 CP, MhV3 RdRp, MhV4 CP and MhV4 RdRp with the most similar sequences deduced by the BLAST search of the NCBI database.

Virus	Virus name	Accession number	Identity (%)	E-Value	Query Coverage (%)
MhV1a/b CP	Thelebolus microsporus totivirus 1 CP	AZT88642.1	49.65	0	89
	Tolypocladium ophioglossoides totivirus 1 CP	AZT88644.1	44.64	0	90
	Beauveria bassiana victorivirus 1 CP	AMQ11130.1	44.00	0	89
	Bipolaris maydis victorivirus 1 CP	AXB26763.1	44.58	0	91
	Helminthosporium victoriae virus 190S CP	NP_619669.2	44.71	0	91
MhV1a/b RdRp	Umbelopsis ramanniana virus 2 RdRp, partial	VFI65724.1	100	0	100
	Thelebolus microsporus totivirus 1 RdRp	AZT88643.1	52.39	0	94
	Tolypocladium ophioglossoides totivirus 1 RdRp	AZT88645.1	48.53	0	94
	Ustilaginoidea virens RNA virus 5 RdRp	YP_009182167.1	47.04	0	100
	Helicobasidium mompa totivirus 1-17 RdRp	NP_898833.1	44.43	0	92
MhV2 CP	Ustilaginoidea virens RNA virus putative CP	YP_007761588.1	40.22	1e-159	90
	Alternaria arborescens victorivirus 1 putative CP	YP_009553477.1	35.91	4e-75	67
	Beauveria bassiana victorivirus 1 CP	AMQ11130.1	33.87	5e-74	69
	Coniothyrium minitans RNA virus putative CP	ALM62230.1	35.54	7e-74	67
	Helicobasidium mompa totivirus 1-17 CP	NP_898832.1	32.78	9e-74	83
MhV2 RdRp	Ustilaginoidea virens RNA virus 1putative RdRp	YP_007761589.1	37.28	2e-160	99
	Diatom colony associated dsRNA virus 11 RdRp	YP_009552795.1	36.03	5e-149	100
	Thelebolus microsporus totivirus 1 RdRp	AZT88643.1	35.43	5e-144	98
	Umbelopsis ramanniana virus 2 RdRp, partial	VFI65724.1	37.28	6e-142	94
	Tolypocladium ophioglossoides totivirus 1 RdRp	AZT88645.1	33.61	1e-134	98
MhV3	Nigrospora oryzae victorivirus 1 CP	YP_009254735.1	36.85	8e-117	93

CP	Alternaria alternata victorivirus 1 CP	BBJ77935.1	34.83	9e-114	94
	Penicillium aurantiogriseum totivirus 1 CP	YP_009212847.1	39.75	2e-113	75
	Alternaria arborescens victorivirus 1 putative CP	YP_009553477.1	39.43	2e-112	74
	Aspergillus homomorphus totivirus 1 CP	AZT88628.1	37.48	4e-112	85
MhV3 RdRp	Diatom colony associated dsRNA virus 10 RdRp	YP_009552793.1	38.91	2e-161	99
	Diatom colony associated dsRNA virus 11 RdRp	YP_009552795.1	37.53	4e-160	97
	Colletotrichum zoysiae totivirus 1 RdRp	AZT88637.1	36.21	6e-156	98
	Umbelopsis ramanniana virus 2 RdRp, partial	VFI65724.1	38.37	2e-154	93
	Diatom colony associated dsRNA virus 7 RdRp	YP_009553338.1	36.15	2e-152	95
MhV4 CP	Trichoderma koningiopsis totivirus 1 CP	QGA70770.1	86.17	0	100
	Wuhan insect virus 26 hypothetical protein 1	YP_009342427.1	51.01	0	78
	Totiviridae sp. hypothetical protein H4Bulk46889_000002, partial	QDH88821.1	41.54	2e-174	96
	Wuhan insect virus 27 hypothetical protein 1	YP_009342433.1	37.86	3e-154	96
	Plasmopara viticola associated totivirus 3 putative CP	QGY72631.1	35.30	2e-138	99
MhV4 RdRp	Trichoderma koningiopsis totivirus 1 RdRp	QGA70771.1	90.35	0	96
	Umbelopsis ramanniana virus 4 RdRp	VUD77425.1	59.69	0	99
	Wuhan insect virus 26 hypothetical protein 2	YP_009342428.1	57.70	0	99
	Wuhan insect virus 27 hypothetical protein 2	YP_009342434.1	52.30	0	100
	Delisea pulchra totivirus IndA RdRp, partial	AMB17470.1	46.75	0	90
