# Supplementary figures and images for: Characterization of Four Novel dsRNA Viruses Isolated from Mucor hiemalis Strains
Source: Viruses. 2021 Nov 21;13(11):2319. doi: 10.3390/v13112319 (PMC8625083; doi:10.3390/v13112319)

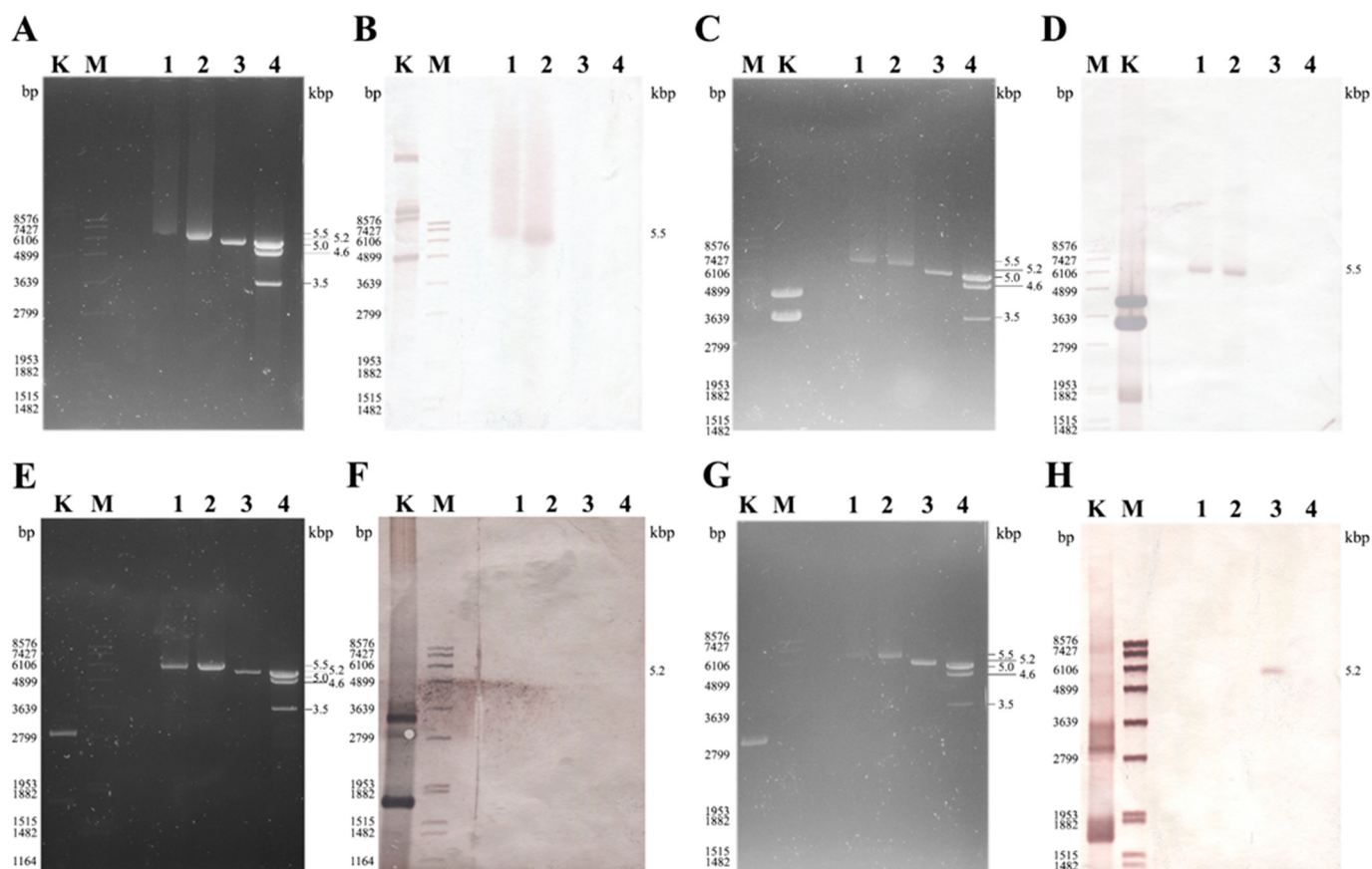

Supplement: Supplementary file 1 [file viruses-13-02319-s001.zip › Supplementary Figure S3.pdf]
